# Urea regulates soil nematode population by enhancing the nematode-trapping ability of nematode-trapping fungi

**DOI:** 10.1038/s41598-024-65167-1

**Published:** 2024-06-21

**Authors:** Zhang Fa, Huang Shuaiyi, Saranyaphat Boonmee, Xiao Wen, Yang Xiaoyan

**Affiliations:** 1 Institute of Eastern-Himalaya Biodiversity Research, Dali University, Dali, 671003 Yunnan China; 2 Center of Excellence in Fungal Research, Mae Fah Luang University, Chiang Rai, 57100 Thailand; 3 School of Science, Mae Fah Luang University, Chiang Rai, 57100 Thailand; 4 Key Laboratory of Yunnan State Education Department On Er’hai Lake Basin Protection and the Sustainable Development Research, Dali University, Dali, 671003 Yunnan China; 5 The Provincial Innovation Team of Biodiversity Conservation and Utility of the Three Parallel Rivers From Dali University, Dali University, Dali, 671003 Yunnan China; 6 International Centre of Biodiversity and Primates Conservation, Dali University, Dali, 671003 Yunnan China

**Keywords:** Ecology, Microbiology

## Abstract

As the most abundant animal in the soil, nematodes are directly or indirectly involved in almost all soil ecological processes. Studying soil nematode population regulation is essential to understanding soil ecological processes. This study found urea combines nematode-trapping fungi to regulate the population of soil nematodes. In soil, compared with no urea, adding 0.2 mg/mL urea after applying *Arthrobotrys oligospora* and *Dactylellina ellipsospora* reduced the number of nematodes by 34.7% and 31.7%. Further, the mechanism of urea couple nematode-trapping fungi to regulate the nematode population was explored in the medium environment. The results showed that the addition of 0.2 mg/ml urea accelerated the trap formation of *A. oligospora* and *D. ellipsosporas* by 50% and 46.5%, and increased the yield of traps of *A. oligospora* and *D. ellipsosporas* by 39.5% and 40.6%, thus, the predatory efficiency of *A. oligospora* and *D. ellipsospora* on nematodes was increased by 34.2% and 32.7%. In conclusion, urea regulates the predation ability of *A. oligospora* and *D. ellipsosporas* to regulate the soil nematode population. This study deepens the understanding of the regulatory pathways of the soil nematodes but also provides a potential new strategy for harmful nematode bio-control.

## Introduction

Soil nematodes are widespread, abundant and highly diverse^1,2^. They can be divided into parasitic nematodes and free-living nematodes (saprophytic and microbivorous), which are dominant in most soil ecosystems with 33–384 species and 3.5–5 million individuals in a square meter of terrestrial soil^3^. They play an essential role in the material and energy cycle of the soil ecosystem due to their high abundance, diversity, and reproductive and metabolic capacity^2,4,5^. Underground, 60%–80% of free-living nematodes feed on soil microorganisms, directly regulating their metabolic characteristics, activity, abundance and driving their colony evolution^6,7^. Aboveground, soil nematodes affect plant growth and directly regulate vegetation characteristics, and the changes in vegetation characteristics react to the underground ecosystem^3,8^. In addition, soil nematodes also directly affect the dynamic balance of soil physical and chemical properties and thus indirectly affect the aboveground and underground soil ecosystems^9^. In brief, soil nematodes are one of the critical junctions for coupling the aboveground and underground parts of terrestrial ecosystems and a critical element for the dynamic balance of the ecosystem.

Studying soil nematode population (SNP) regulation is vital in understanding soil ecological processes^10,11^. Soil abiotic factors play a crucial role in regulating SNP, among which nitrogen (N) is considered one of the most critical factors^12,13^. Previous studies have shown that N addition will generally reduce the richness and diversity of soil nematodes, but nematodes with different lifestyles responded differently to N addition. For example, in the forest, grassland and farmland ecosystems, with the addition of N, the number of root herbivores, fungivores and omnivores-predators decreased, while the number of opportunistic bacterivores increased^14–16^. In addition, the response of SNP to N addition was also different in different ecosystems. For example, adding 120 kg N ha^-1^ yr^-1^ in the semi-arid grassland ecosystem suppressed SNP^17^. Whereas, in subtropical acidic soils of China, N addition increased SNP^18^. The changes in soil pH, aboveground vegetation characteristics and ammonium toxicity caused by N addition are generally considered the main pathways of SNP regulation^12,19^. These three SNP regulation pathways induced by N addition usually require a longer time or high level of N addition to manifest their effects. However, these three regulation pathways could not solve the controversy over the response range and even direction of SNP to N addition, and it was even more challenging to explain the changes of SNP in the short term after the addition of low N concentration^20^. Therefore, we speculated that there might be other unknown regulatory pathways besides the three regulatory pathways mentioned above after N addition.

Nematode-trapping fungi (NTF) are a group of fungi that can produce special mycelium structures to capture nematodes^21,22^. Their ability to feed on nematodes is the product of adaptive evolution in response to N deficiency in the soil habitat^23,24^. These fungi are widely distributed in various soil habitats and are an important balancing factor for SNP in nature^25^. In the course of studying these group fungi, we found that urea can change the predation characteristics of these group fungi. Therefore, we hypothesized that adding urea to the soil would drive NTF to regulate SNP. In order to verify this hypothesis, the combined regulation of urea and NTF on SNP was investigated in soil and medium environments. The results further enhance our understanding of the complexity and diversity of SNP regulation pathways and provide a potential new strategy for harmful nematode control.

## Results

### Effect of urea on the ability of NTF to regulate soil nematode population (SNP)

After testing, two datasets of soil nematode quantity after adding different concentrations of urea after applying *A. oligospora* and *D. ellipsospora* did not satisfy normal distribution (*P* = 0.012, *P* = 0.0003), and the latter did not satisfy variance homogeneity either (*P* = 0.033). After the reciprocal conversion of the original datasets, the datasets of *A. oligospora* and *D. ellipsospora* are consistent with normal distribution (*P* = 0.143, *P* = 0.323) and homogeneity of variance (*P* = 0.503, *P* = 0.810). One-way ANOVA of the converted datasets showed that there are significant differences between different groups of the datasets of *A. oligospora* (F (6, 42) = 340, *P* < 0.0001) and *D. ellipsospora* (F (6, 42) = 352.8, *P* < 0.0001).

**Figure 1:**
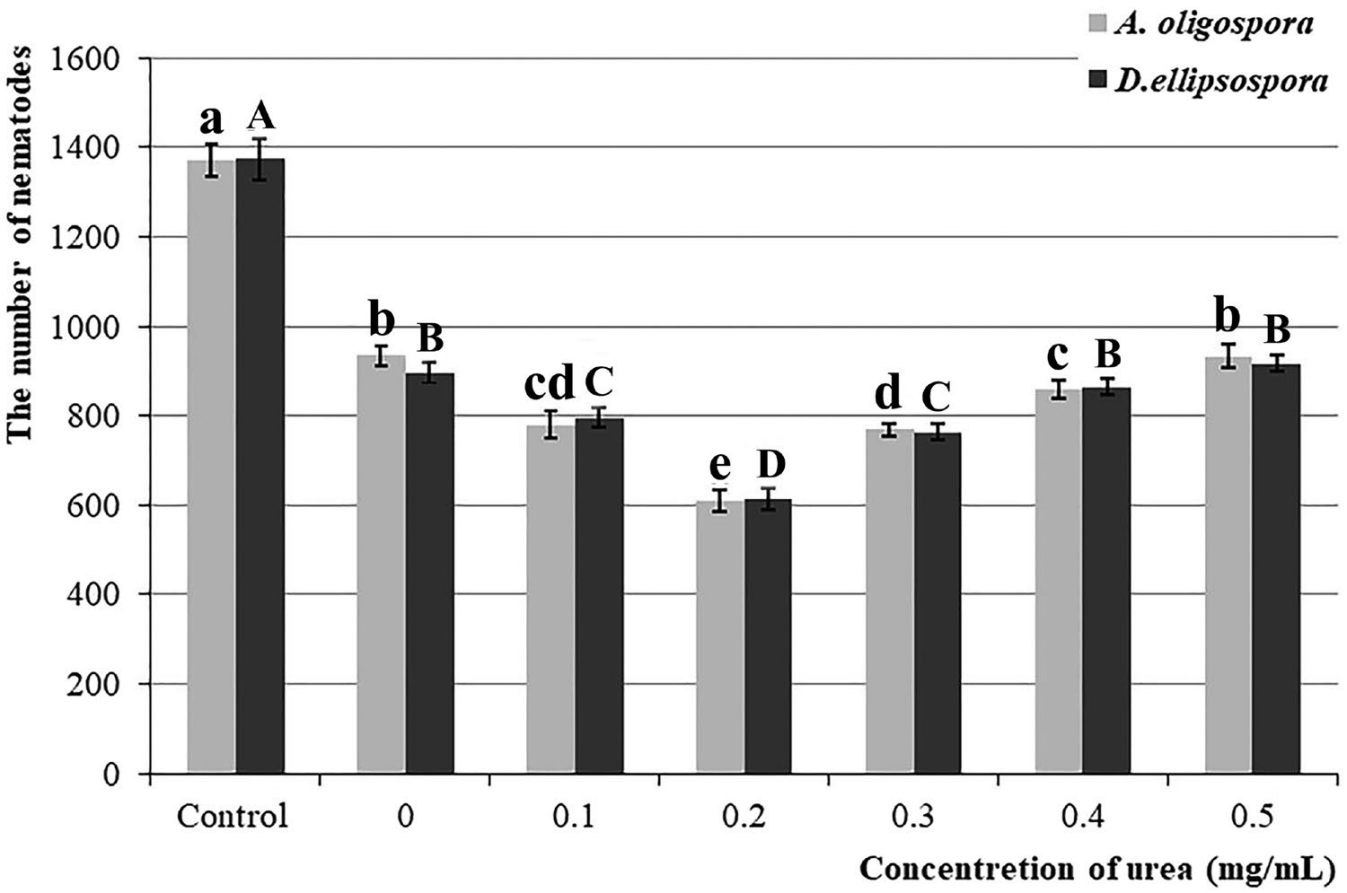
The effects of different concentrations of urea on the number of soil nematodes. Values are the mean ± SD (n = 7). The lowercase and uppercase letters indicate significant differences among different urea concentrations combined with *Arthrobotrys* *oligospora* and *Dactylellina* *ellipsospora*, respectively.

Compared with the control group, the application of *A. oligospora* and *D. ellipsospora* alone decreased the number of soil nematodes by 31.8% (1371.7 vs. 935.6 nematodes, *P* < 0.0001) and 34.8% (1373.9 vs. 896.1 nematodes, *P* < 0.0001), respectively. The experimental groups showed that the addition of certain concentrations of urea could significantly reduce the number of nematodes after the application of *A. oligospora* and *D. ellipsospora*: when the urea concentration was 0–0.2 mg/mL, its reducing effect on the number of nematodes gradually strengthened with increasing urea concentration. When the concentration was 0.2 mg/mL, the number of nematodes in the soil was the lowest, at an average of 610.6 (*A. oligospora*) and 612.4 (*D. ellipsospora*), which was a decrease of 34.7% (*P* < 0.0001) and 31.7% (*P* < 0.0001) compared with the 935.6 and 896.1 nematodes, respectively when no urea was added. When the urea concentration was more than 0.2 mg/mL, the effect of increasing the urea concentration on reducing the nematode population gradually weakened (Fig. 1).

### Trap formation rate and yield of NTF under different urea concentrations

The normal distribution and variance homogeneity test showed that the datasets of the trap formation rates and yield of *A. oligospora* and *D. ellipsospora* under different urea concentrations met the normal distribution and variance homogeneity (all P values exceeded 0.05). The results of one-way ANOVA showed that there are significant differences in two datasets of trap formation rates of *A. oligospora* (F (5, 24) = 73.5, *P* < 0.0001) and *D. ellipsospora* (F (5, 24) = 44, *P* < 0.0001) under different urea concentrations. There also are significant differences in the datasets of trap yield of *A. oligospora* (F (5, 24) = 83.05, *P* < 0.0001) and *D. ellipsospora* (F (5, 24) = 49.85, *P* < 0.0001).

**Figure 2:**
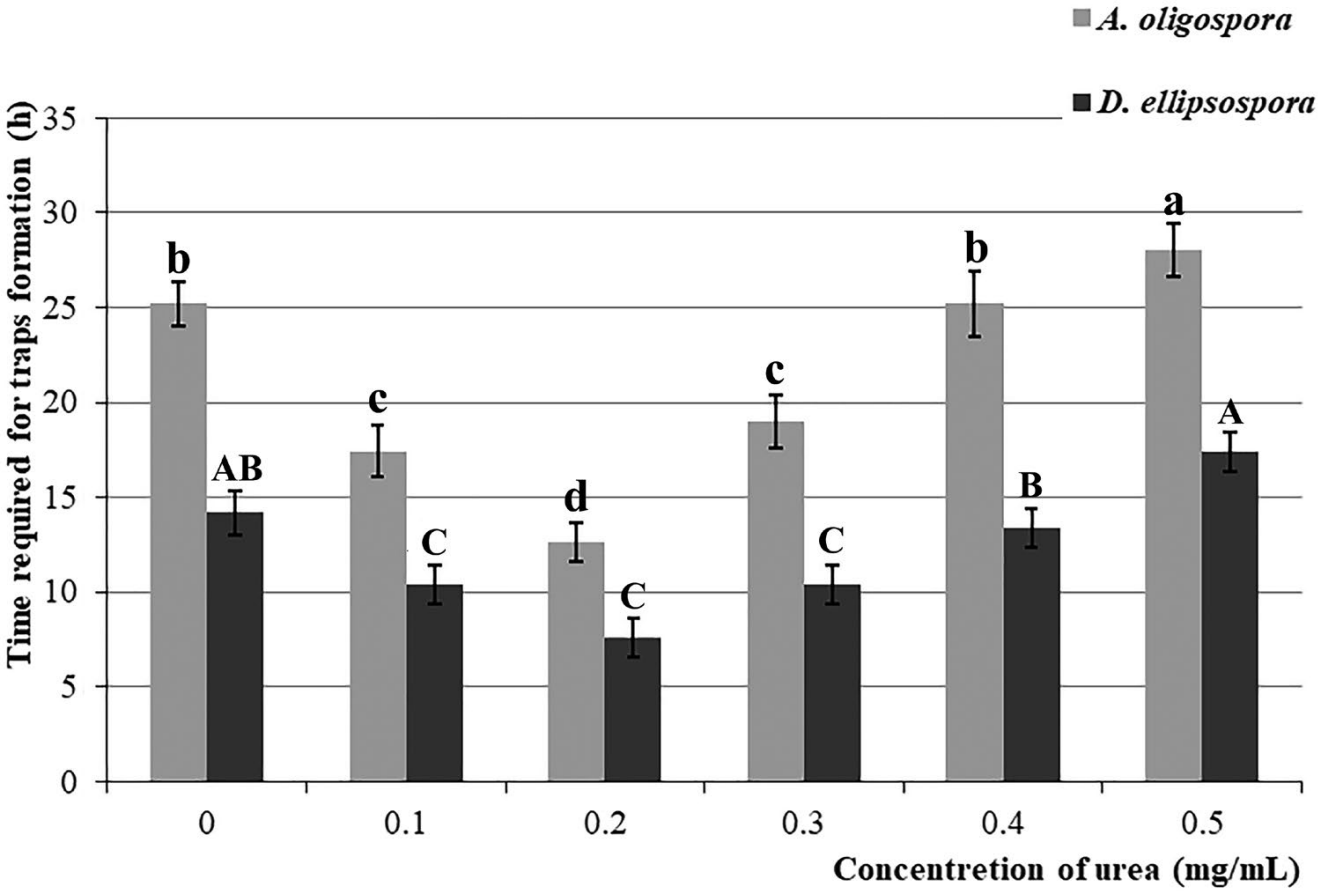
Required time for trap formation of *Arthrobotrys* *oligospora* and *Dactylellina* *ellipsospora* under different urea concentrations. Values are the mean ± SD (n = 5). The lowercase and uppercase letters indicate significant differences among different urea concentrations combined with *Arthrobotrys* *oligospora* and *Dactylellina* *ellipsospora*, respectively.

**Figure 3:**
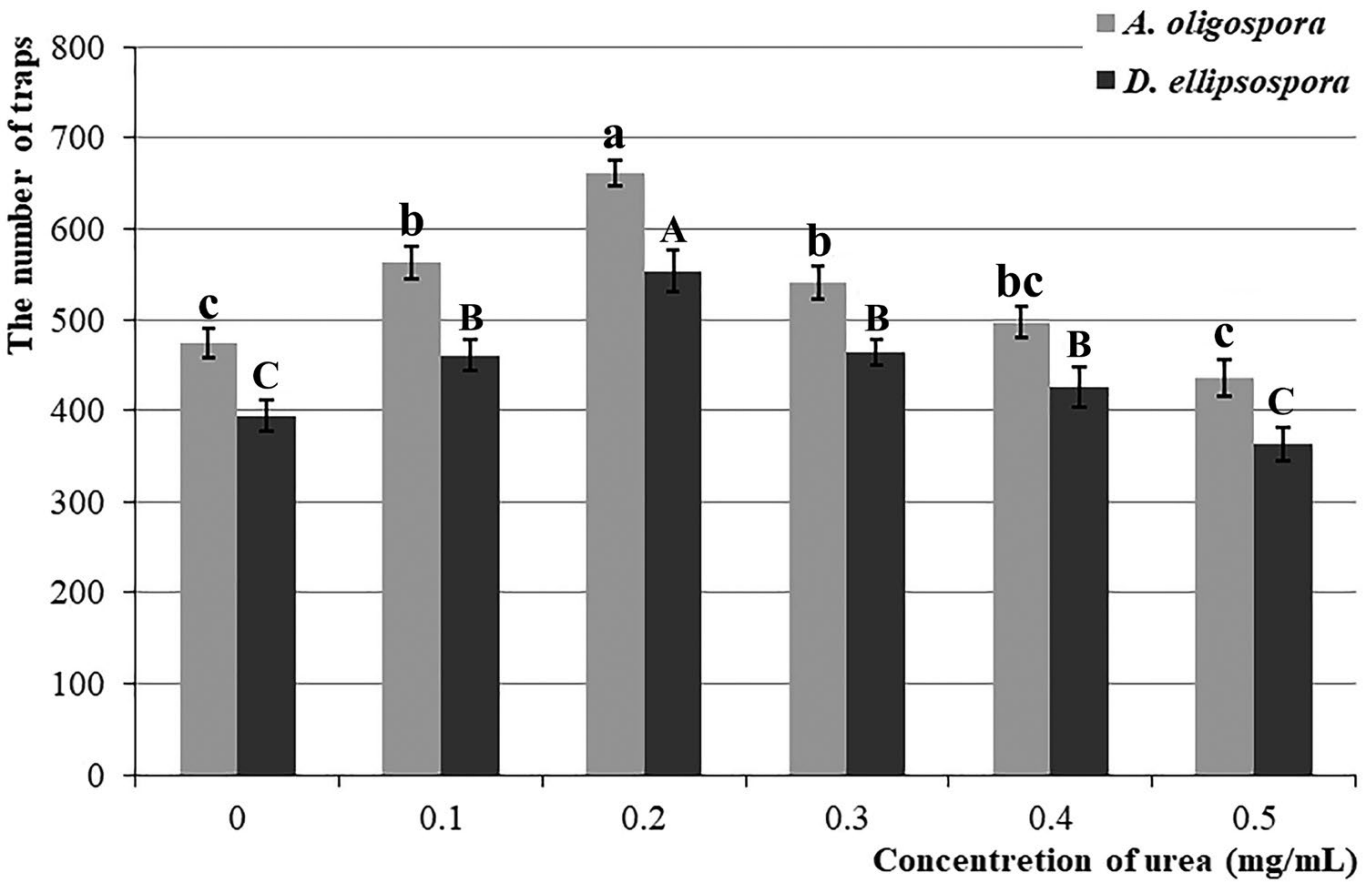
The number of traps after 72 h of *Arthrobotrys* *oligospora* and *Dactylellina* *ellipsospora* under different urea concentrations. Values are the mean ± SD (n = 5). The lowercase and uppercase letters indicate significant differences among different urea concentrations combined with *Arthrobotrys* *oligospora* and *Dactylellina* *ellipsospora*, respectively.

When the urea concentration was in the range of 0–0.2 mg/mL, with increasing urea concentration, the effect of promoting NTF to produce traps was gradually strengthened. When the urea concentration was 0.2 mg/mL, *A. oligospora* and *D. ellipsospora* required the shortest time to produce traps (they took 12.6 and 7.6 h on average, which was reduced by 50% (*P* < 0.0001) and 46.5% (*P* < 0.0001) compared with the 25.2 and 14.2 h, respectively when no urea was added (Fig. 2). In addition, at this urea concentration, *A. oligospora* and *D. ellipsospora* had the highest trap yield with an average of 662.2 and 553.8 traps, which was 39.5% (*P* < 0.0001) and 40.6% (*P* < 0.0001) higher than the 474.6 and 393.8 traps, respectively when no urea was added. When the urea concentration exceeded 0.2 mg/mL, the trap formation time and trap yield of *A. oligospora* and *D. ellipsospora* gradually lengthened (Fig. 3).

### Nematocidal ability of NTF under different urea concentrations

**Figure 4:**
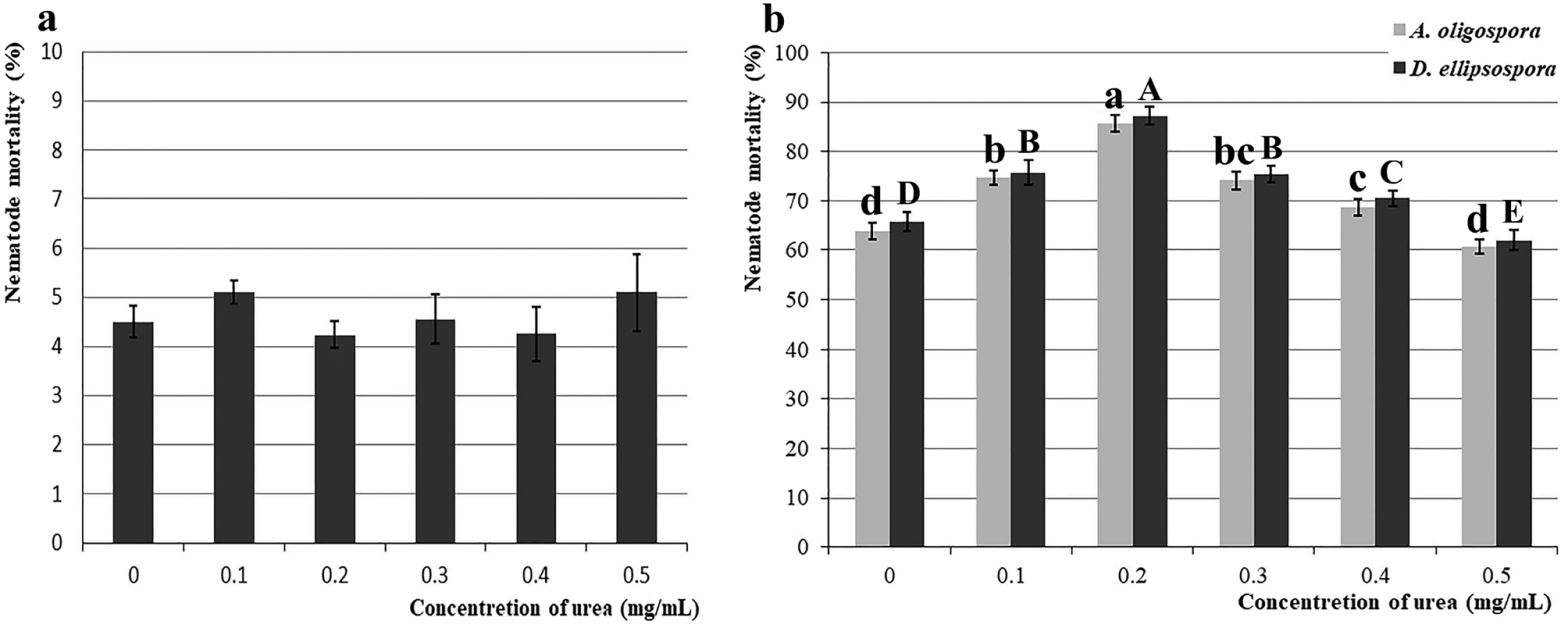
Nematode mortality under different urea concentrations. (**a**), nematode mortality under different concentrations of urea alone; (**b**), Nematode mortality of *Arthrobotrys* *oligospora* and *Dactylellina* *ellipsospora* under different urea concentrations. Values are the mean ± SD (n = 5). The lowercase and uppercase letters indicate significant differences among different urea concentrations combined with *Arthrobotrys* *oligospora* and *Dactylellina* *ellipsospora*, respectively.

The test results showed that the dataset of nematode mortality under different urea concentrations is consistent with normal distribution (*P* = 0.284) and homogeneity of variance (*P* = 0.314). The one-way ANOVA result showed no significant difference in the dataset of nematode mortality under different concentrations of urea alone (F (5, 24) = 2.052, *P* = 0.1072). Therefore it was concluded that adding urea alone had no obvious poisoning effect on the nematodes (Fig. 4a).

After the normal distribution and variance homogeneity test, the datasets of nematode mortality of *A. oligospora* and *D. ellipsospora* under different urea concentrations are consistent with normal distribution (*P* = 0.142, *P* = 0.180) and homogeneity of variance (*P* = 0.999, *P* = 0.757). One-way ANOVA showed that there are significant differences in two datasets of nematode mortality of *A. oligospora* (F (5, 24) = 114.4, *P* < 0.0001) and *D. ellipsospora* (F (5, 24) = 85.7, *P* < 0.0001) under different urea concentrations.

In the experimental group, with increasing urea concentrations, nematode mortality gradually increased. When the urea concentration reached 0.2 mg/mL, the nematode mortality of *A. oligospora* and *D. ellipsospora* was the highest, at an average of 85.6% and 87.2%, which was an increase of 34.2% (*P* < 0.0001) and 32.7% (*P* < 0.0001) over the 63.8% and 65.7% respectively when no urea was added. When the urea concentration exceeded 0.2 mg/mL, the promotion effect of the increase in urea concentration on the predation ability of *A. oligospora* and *D. ellipsospora* was weakened (Fig. 4b).

## Discussion

In this study, the number of nematodes in soil decreased by 31.8% and 34.8% after *A. oligospora* and *D. ellipsospora* were added alone (Fig. 1), emphasizing the significance of nematode-trapping fungi (NTF) in soil nematode population (SNP) regulation. After adding *A. oligospora* and *D. ellipsospora*, the density of nematodes was further changed by adding different urea concentrations. The addition of 0.2 mg/ml of urea further reduced the number of nematodes by nearly 30% (Fig. 1), indicating that different urea concentrations could regulate the regulation ability of NTF to SNP, and this pathway is an important way for SNP regulation. In the medium environment, the addition of low concentration urea (0.1–0.4 mg/mL) accelerated the formation and increased the yield of the trapping structure, then improved the ability of *A. oligospora* and *D. ellipsospora* to catch nematodes. In comparison, the addition of high concentration urea (0.5 mg/mL) inhibited the formation of the trapping structure of *A. oligospora* and *D. ellipsospora* (Figs. 2, 3, 4). The results confirmed that urea regulates SNP by regulating the predation ability of NTF.

The molecular mechanism by which urea regulates the insecticidal ability of NTF may be similar to the mechanism by which soil bacteria mobilize NTF to capture nematodes: certain soil bacteria release urea to the surrounding environment when they experience predation stress from nematodes, and urea enters NTF cells under urea transporters and decomposes into ammonium ions under the action of urease. As a signal factor, ammonium ions directly promote NTF to produce traps quickly to catch nematodes to protect bacterial populations from nematodes^26^. According to this mechanism, it can be inferred that applying a specific concentration of ammonium salt may also have a similar effect as urea. Even because ammonium salt eliminates the step of urease decomposition, it can more quickly act on NTF cells, and the effect of promoting NTF to catch nematodes may be better and faster than that of urea. Except for NTF, there are other nematophagous microorganisms in the soil to balance SNP, such as endoparasitic fungi, egg parasitic fungi and nematophagous bacteria^27–29^. Similar to NTF, the ability to feed on nematodes of these nematophagous microorganisms is thought to be the product of adaptive evolution in response to N deficiency in the soil habitat^28–30^, the change of N in soil may also change their nematocidal function, and then regulate SNP. Therefore, the combined regulatory pathways of N and these microorganisms should be emphasized in future SNP regulation studies. In addition, other soil abiotic factors besides N (such as carbon, organic matter, temperature and precipitation, etc.) also affect the species composition, richness and insecticidal characteristics of nematophagous microorganisms^31,32^, thus affecting the balance of SNP. Therefore, future studies should consider the combined regulatory pathways of these soil abiotic factors and nematophagous microorganisms.

Our study also provides a potential new strategy for soil harmful nematode control. Plant-parasitic nematodes are serious plant pests worldwide, causing crop-related economic losses of more than $100 billion yearly^33,34^. Although chemical insecticides or repellents such as Temik and Aldicarb are effective in controlling these diseases, they not only leave pesticide residues and cause ecological damage but also increase the resistance of nematodes, forming a vicious cycle^35,36^. Therefore, using NTF to control them is the most promising method^37^. However, in previous studies, most of the studied strains showed excellent nematicidal effects in pure culture, whereas their field experiment tests were unsatisfactory^38,39^. Improving the effectiveness of these preparations is the greatest challenge of applying NTF. In this study, the addition of a proper concentration of urea can significantly improve the predation efficiency of NTF on nematodes, whether in the culture medium or field soil environment (Figs. 2, 3, 4). In addition, as one of the most commonly used N fertilizers in agricultural production, urea has the advantages of low costs and nontoxic side effects. Therefore, adding urea after an NTF application can significantly improve the control effect of NTF on nematodes without greatly increasing control costs. The combined application of urea with NTF is an effective and feasible nematode control strategy.

## Methods

### Experimental materials

**Figure 5:**
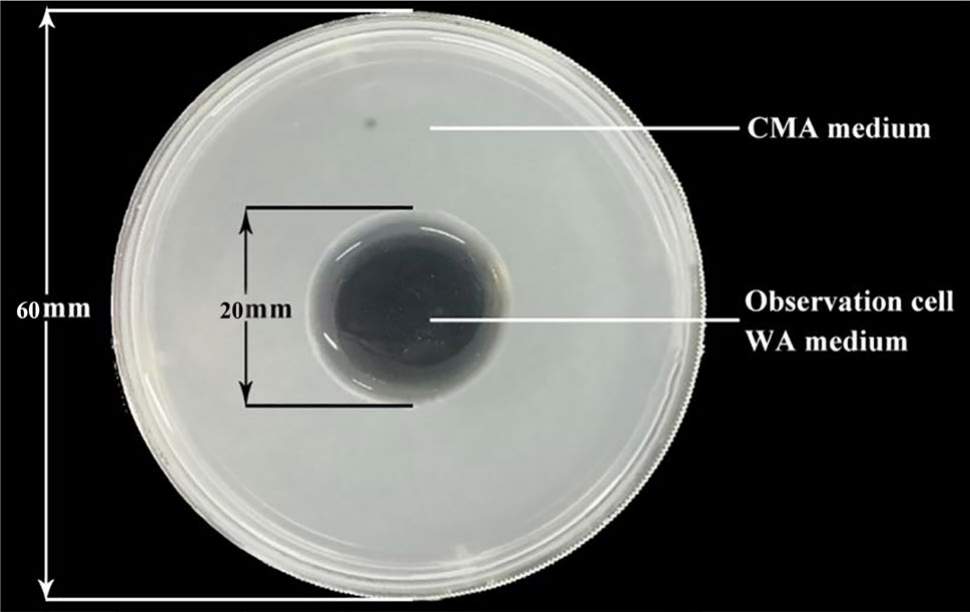
CMA observation plate.

Corn meal agar (CMA) observation plate: A 20 mm diameter hole was punched in the center of the CMA plate^22^ with a sterile punch, and approximately 0.8 mL sterilized water agar (WA) medium^22^ was injected into the hole with a sterile syringe (Fig. 5).

Urea solution: An appropriate amount of urea was weighed and prepared with sterile water to form solutions with concentrations of 0, 0.1, 0.2, 0.3, 0.4 and 0.5 mg/mL. The solution was filtered using a filter membrane with an aperture of 0.22 μm and stored at 4 °C.

Bait nematodes: *Panagrellus redivivus* Goodey (a free-living soil nematode that is often used as a target organism in nematode control research)^22,40,41^ was provided by Germplasm Resources Center of Institute of Eastern-Himalaya Biodiversity Research. The nematodes were separated from the oatmeal medium using the Baermann funnel method^42^. The nematode concentration was counted by a stereo microscope and adjusted to 5 × 10^3^/mL with sterile water.

Testing NTF strain: *Arthrobotrys oligospora* Fres and *Dactylellina ellipsospora* Preuss (Orbiliaceae, Orbiliomycetes) catch nematodes using adhesive networks and adhesive knobs, respectively^22^. They were identified and provided by the Germplasm Resources Center of the Institute of Eastern-Himalaya Biodiversity Research. The strain was transferred to fresh CMA plates and incubated at 27 °C for 8–15 d. Sterile water (5–6 mL) was added to the plates, the conidia of *A. oligospora* and *D. ellipsospora* were eluted, and spore eluent was collected, respectively. The spore concentration was counted by cell-count boards under microscope and adjusted to 10^4^/mL with sterile water.

Testing soil sample: The field soil containing a large number of nematodes was collected from the potato planting field in the Agronomy Practice and Teaching Base of Dali University. The large particles in the soil were sieved out, and the remaining soil was mixed. Ninety-eight soil samples (each sample weighed 2 kg) were weighed and placed into 98 flowerpots with a diameter of 38 cm. All pots were placed in a cool area, and 100 mL of sterile water was poured on them every other day to keep the soil moist. From Day 3 onwards, the nematode density of each sample was monitored every two days with the improved Baermann funnel method^42^, until the density of nematodes was stable three times (the 11th day). Then, follow-up experiments were carried out.

### Synergistic effect of urea and NTF on soil nematode population (SNP)

The 98 pots of soil with stable nematode densities were randomly divided into 2 groups (49 pots per group) for the experiments of *A. oligospora* and *D. ellipsospora*, respectively. The following experimental methods are as follows (taking the experiment of *A. oligospora* as an example, the experiment of *D. ellipsospora* is the same).

The 49 pots of soil were randomly divided into 7 groups (7 replicates per group): 1 control group and 6 experimental groups. The control group received no treatment. For the experimental groups, 30 ml spore eluent of *A. oligospora* was evenly added to each pot. Then, from day 18, the nematode density of each pot was monitored every other day with the improved Baermann funnel method^42^ until the density of nematodes was stable three times (the 28th day). 200 ml of urea solution at 6 concentrations was evenly added to each pot of the 6 experimental groups. After 18 days, one 100 g soil sample was collected evenly from each pot in the control and experimental groups (a total of 7 soil samples per treatment). The nematodes in each sample were separated and counted using the improved Baermann funnel method^42^. During the experiment, the pots were placed in a cool area, and 100 ml of sterile water was poured into each pot every other day to keep the soil moist.

### Effect of urea on the traps formation rate and yield of NTF

Taking the experiment of *A. oligospora* as an example, the experiment of *D. ellipsospora* is the same.

Five hundred microlitres of spore eluent of *A. oligospora* was evenly spread onto thirty CMA observation plates and incubated at 27 °C. When the mycelium overspread the observation cell, approximately 500 nematodes (*P. redivivus*) were added to the observation cell of each plate. Thirty plates were randomly divided into 6 groups and marked as 0, 0.1, 0.2, 0.3, 0.4, and 0.5 (each group contained 5 replicates). Six concentrations of urea were added to the observation cell of the corresponding observation plates (150 μL was added to each plate). The formation of traps was observed every hour, and the earliest formation time was recorded. After 48 h, the number of traps in the observation cell was counted.

### Effect of urea on the nematocidal ability of NTF

Taking the experiment of *A. oligospora* as an example, the experiment of *D. ellipsospora* is the same.

Control group: The urea and nematodes were transferred to fresh CMA observation plates according to the above methods to eliminate the toxic effect of urea on nematodes.

Experimental group: The spore eluents of *A. oligospora*, urea and nematodes were transferred to CMA observation plates according to the above methods. After 96 h, the dead nematodes and live nematodes in the observation cells of each plate in the control and experimental groups were counted under stereoscopic microscope, and nematode mortality was calculated.

## Data management and analysis

We calculated the nematode mortality as the following formula: Nematode mortality = Number of dead nematodes/(number of dead nematodes + number of live nematodes) × 100%.

Through the above experiments, nine datasets were obtained: 1, 2) The number of soil nematodes after adding different concentrations of urea after applying *A. oligospora* and *D. ellipsospora*. 3,4) The required time for traps formation of *A. oligospora* and *D. ellipsospora* under different concentrations of urea. 5, 6) The traps yields of *A. oligospora* and *D. ellipsospora* under different concentrations of urea. 7, 8) The nematode mortality of *A. oligospora* and *D. ellipsospora* under different concentrations of urea. 9) The nematode mortality under different concentration of urea alone. Excel (2010) was used to manage the experimental data and draw the bar charts with mean values and standard deviation (sd).

In order to clarify the effect of urea concentration on soil nematode quantity after application of *A. oligospora* and *D. ellipsospora* and the effect of urea concentration on the trapping function of *A. oligospora* and *D. ellipsospora*. The differences between the control and treatment groups in each dataset were compared in pairs, respectively. The detailed methods are as follows (taking the number of soil nematodes after adding different concentrations of urea after applying *A. oligospora* as an example): 1) SPSS (version 20; SPSS Inc., USA) was used to perform the Shapiro–Wilk test to check the normality of data distribution. When the *P* value exceeds 0.05, the dataset conforms to the normal distribution and vice versa. 2) One-way ANOVA test in SPSS (version 20; SPSS Inc., USA) was used to test the variance homogeneity of the dataset. When the *P* value exceeds 0.05, the dataset conforms to variance homogeneity and vice versa. 3) For a dataset that satisfies both normal distribution and homogeneity of variance, the one-way ANOVA was performed using SPSS (version 20; SPSS Inc., USA) to clarify the differences in the dataset. The Tukey Honest Significance Difference test (HSD, 5% precision level) in SPSS (version 20; SPSS Inc., USA) was used further to determine the pairwise differences between treatment and control groups. If the dataset does not meet the normal distribution or homogeneity of variance, log or reciprocal conversion is performed on the dataset using the data conversion function in SPSS (version 20; SPSS Inc., USA), and steps 1 and 2 are repeated with the converted dataset. If the converted dataset meets the normal distribution and homogeneity of variance, the one-way ANOVA was performed. The HSD test at 5% precision level was used to ascertain the significance and non-significance of different treatment and control groups.

## Data Availability

The data that support the findings of this study are contained within the article.
